# Ocular Manifestations of Flavivirus Infections

**DOI:** 10.3390/pathogens12121457

**Published:** 2023-12-15

**Authors:** Sourour Meziou Zina, Gautier Hoarau, Marc Labetoulle, Moncef Khairallah, Antoine Rousseau

**Affiliations:** 1 Department of Ophthalmology, Bicêtre Hospital, Public Assistance, Hospitals of Paris, Reference Network for Rare Diseases in Ophthalmology (OPHTARA), 94270 Le Kremlin-Bicêtre, France; sourour.meziou@aphp.com (S.M.Z.); drgautierhoarau@gmail.com (G.H.); marc.labetoulle@aphp.fr (M.L.); 2 Department of Ophthalmology, Faculty of Medicine, University of Monastir, Monastir 5019, Tunisia; moncef.khairallah@rns.tn; 3 Center for Immunology of Viral, Auto-Immune, Hematological and Bacterial Diseases (IMVA-HB), Infectious Diseases Models for Innovative Therapies (IDMIT), French Alternative Energies and Atomic Commission (CEA), 92260 Fontenay-aux-Roses, France

**Keywords:** flavivirus, eye, West Nile virus, dengue fever virus, yellow fever, Zika virus, Japanese encephalitis virus, Kyasanur forest disease virus

## Abstract

Flaviviruses are a group of positive-sense, single-stranded RNA viruses predominantly transmitted by arthropods (mainly mosquitoes) that cause severe endemic infections and epidemics on a global scale. They represent a major cause of systemic morbidity and death and are expanding worldwide. Among this group, dengue fever, the West Nile virus, yellow fever, Japanese Encephalitis, and, recently, the Zika virus have been linked to a spectrum of ocular manifestations. These manifestations encompass subconjunctival hemorrhages and conjunctivitis, anterior and posterior uveitis (inclusive of vitritis, chorioretinitis, and retinal vasculitis), maculopathy, retinal hemorrhages, and optic neuritis. Clinical diagnosis of these infectious diseases is primarily based on epidemiological data, history, systemic symptoms and signs, and the pattern of ocular involvement. Diagnosis confirmation relies on laboratory testing, including RT-PCR and serological testing. Ocular involvement typically follows a self-limited course but can result in irreversible visual impairment. Effective treatments of flavivirus infections are currently unavailable. Prevention remains the mainstay for arthropod vector and zoonotic disease control. Effective vaccines are available only for the yellow fever virus, dengue virus, and Japanese Encephalitis virus. This review comprehensively summarizes the current knowledge regarding the ophthalmic manifestations of the foremost flavivirus-associated human diseases.

## 1. Introduction

Flaviviruses constitute a group of 90 positive-sense, single-stranded RNA viruses, with 30 capable of causing severe disease in both humans and animals. This viral family is primarily transmitted by hematophagous arthropods, notably ticks and mosquitoes, resulting in widespread endemic infections and global epidemics [1,2]. Some members of the flavivirus family continue to pose a significant global threat, leading to systemic morbidity and an expanding worldwide mortality [3]. Flaviviral infections often manifest as either asymptomatic or non-specific febrile illnesses. However, severe and potentially fatal systemic complications, such as hemorrhagic fever and neurological involvement, can occur. In addition to systemic infections that entail hemorrhages, vascular leakage, encephalitis, microcephaly, and Guillain–Barre syndrome, certain specific flaviviruses, including dengue fever (DFV), West Nile virus (WNV), yellow fever virus (YFV), Japanese Encephalitis virus (JEV), and Zika virus, have recently been linked to uveitis and other ocular manifestations [4,5,6,7,8]. There are no specific antiviral therapies for flavivirus infections and treatment is essentially symptomatic [3,4,5,7]. This manuscript aims to provide an overview of the ophthalmic manifestations associated with the most significant flavivirus-related human diseases.

## 2. General Aspects

### 2.1. Virological Features

**Figure 1:**
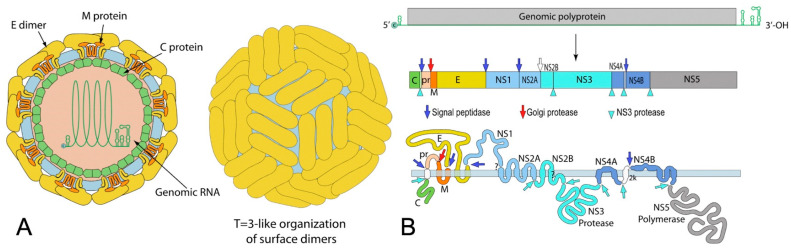
Schematic representation of a flavivirus viral particle (**A**) and genome (**B**). Adapted from https://viralzone.expasy.org/24 (accessed on 1 January 2011) with authorization [12].

Flaviviruses are a family of enveloped viruses with a compact, positive-sense, single-stranded RNA genome of approximately 10.5 kb (Figure 1). This genome encodes three structural and seven non-structural proteins. These ten essential proteins serve crucial functions in virion assembly, cell receptor binding and entry, viral polyprotein processing, and viral replication. After entering the host via an infected vector, the virus infects macrophages, monocytes, and dendritic cells [9,10,11]. Flaviviruses enter cells through receptor-mediated endocytosis, binding with host endosomes in an acidic environment, leading to conformational changes in their envelope (E) glycoprotein [2]. These changes facilitate fusion of the host and viral membranes, releasing the nucleocapsid and viral RNA genome into the host cell’s cytoplasm. The polypeptide is then translated into the ten viral proteins, comprising three structural proteins (C, prM, and E protein) and seven non-structural proteins (NS1, NS2A, NS2B, NS3, NS4A, NS4B, and NS5) [1,2]. The non-structural proteins oversee viral genome replication, budding, and the hijacking of host cell machinery. Following translation, RNA-dependent RNA polymerase (RdRp), NS5, generates a negative strand from genomic RNA, serving as a template for the production of a new positive strand. In the rough Endoplasmic Reticulum (ER), viral proteins commence assembly, packaging viral RNA with C, E, and prM structural proteins. Viral particles are subsequently transported to the trans-Golgi network, where prM is cleaved into M. The mature virus is then released from the host into the extracellular space through exocytosis [2].

### 2.2. Transmission

Flaviviruses persist in an enzootic cycle, primarily involving mosquitoes as the main vectors and mammals and birds as the amplifying hosts, and are incidentally transmitted to humans [3]. While arthropod-borne transmission is predominant, alternative transmission routes have been documented. For instance, WNV can be transmitted among humans via blood transfusions and organ transplants, or from mother to newborn through transplacental transmission [13,14,15]. WNV can also be transmitted orally in hamsters, birds, and mice [16,17,18]. Some cases of human-to-human DENV transmissions via blood transfusions have been reported [19,20]. JEV and Zika virus can be transmitted transplacentally from an infected mother to the fetus in the first and second trimester [21,22]. Seminal transmission of JEV in pigs, resulting in embryo abortion, has been documented [23]. Similarly, ZIKV has been shown to persist in bodily fluids, suggesting a route of horizontal transmission [24].

### 2.3. Diagnosis

The diagnosis of flavivirus infections is typically carried out using conventional methods, including serology and molecular assays [25]. Reverse Transcription Polymerase Chain Reaction (RT-PCR) is commonly employed during the early stage of the disease, typically within the first week of infection. Serological testing, specifically Enzyme-Linked Immunosorbent Assays (ELISAs), is generally used during the acute phase to detect IgM antibodies and in the later stages to detect IgG antibodies. However, it is important to note that cross-reactivity is common among viruses in the *Flaviviridae* family, which can reduce the specificity of the diagnosis [25]. In this context, a specific technique of ELISA called “antibody capture ELISA”, also known as “sandwich ELISA”, has been developed to enhance the accuracy of the assay [26]. Moreover, the plaque reduction neutralization test (PRNT), often considered as a gold standard, is used to confirm equivocal results. The PRNT is highly specific among serological assays, showing minimal cross-reactivity, and is used to detect neutralizing antibodies [25]. Finally, cell culture procedures may be conducted at designated research facilities, aimed at isolating the viral strain. Handling of the isolated virus should take place in biosafety level 3 laboratories, especially in the case of JEV [25,27].

## 3. Ocular Complications of Flaviviruses

### 3.1. Dengue Fever (DF)

#### 3.1.1. Epidemiology

Dengue virus comprises four distinct serotypes (DF-1 to DF-4), each lacking cross-immunity, thus allowing for multiple dengue fever infections in a given individual [28]. Transmission occurs via the bite of an infected female *Aedes aegypti/albopictus* mosquito. *Aedes albopictus* vectors tend to trigger slower outbreaks compared to the rapid epidemics associated with *Aedes aegypti* [29]. DF ranks as a significant arthropod-borne disease in tropical and subtropical regions, endemic in over 100 countries spanning America, Southeast Asia, the Western Pacific, Africa, and the Eastern Mediterranean [30]. The incidence of DF has surged 30-fold in recent decades, affecting an estimated 390 million people annually [5]. This has resulted in approximately 96 million symptomatic infections yearly, with two million cases of severe dengue and an annual death toll of 21,000 [31,32,33]. Manifesting in endemic–epidemic cycles within densely populated tropical urban areas, the majority of infections occur in children across Asia and young adults within the American tropics, though the impact extends to other continents [34].

#### 3.1.2. Systemic Manifestations

Dengue fever occurs 3–14 days after a mosquito bite [28]. Infection can be asymptomatic, result in a non-specific febrile illness, or exhibit classic DF symptoms, including a sudden high fever, a severe headache, myalgias, arthralgias, nausea, vomiting, hepatomegaly, lymphopenia, and a maculopapular rash. Most DF cases resolve spontaneously. However, a minority of patients may progress to a life-threatening condition known as dengue hemorrhagic fever (or dengue shock syndrome), characterized by thrombocytopenia, hypotension, and the potential for multi-system organ failure [5].

#### 3.1.3. Ocular Manifestations

The precise incidence of ocular manifestations during DF remains uncertain since most studies focus on cases seen by hospital specialists. Nevertheless, reported incidences range from 7.1% to 40.3%, likely reflecting varying disease severities and the diverse ocular assessments used in each study [35,36]. Ocular involvement in DF, typically bilateral, can result from thrombocytopenia, inflammation, and ischemic mechanisms [5,37]. These ocular manifestations can occur from days to months after the onset of fever.

**Figure 2:**
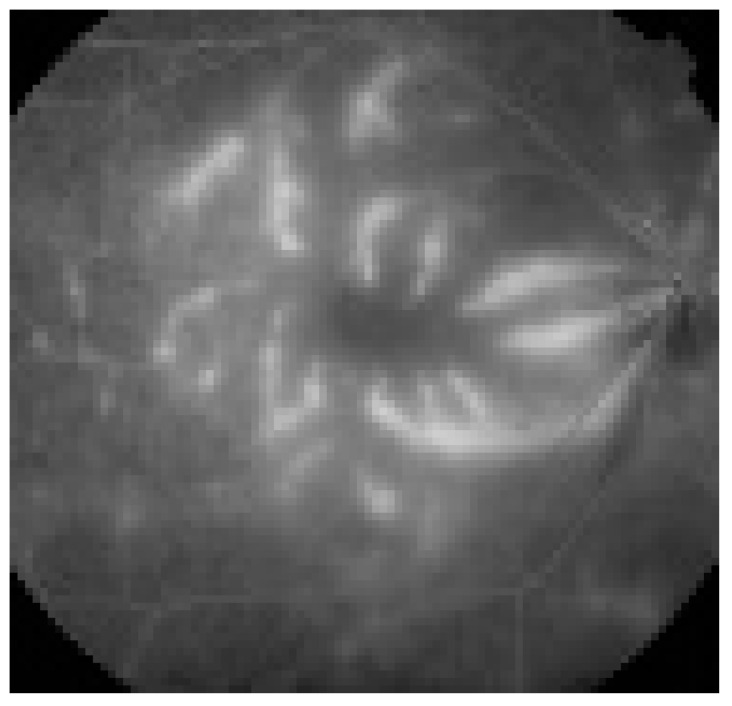
Fluorescein angiogram of the right eye of a 33-year-old woman presenting with sudden loss of vision 1 week after the onset of dengue hemorrhagic fever showing dengue maculopathy, manifesting as severe retinal vasculitis with prominent vascular leakage (Courtesy of Pr Soon Phaik Chee).

**Figure 3:**
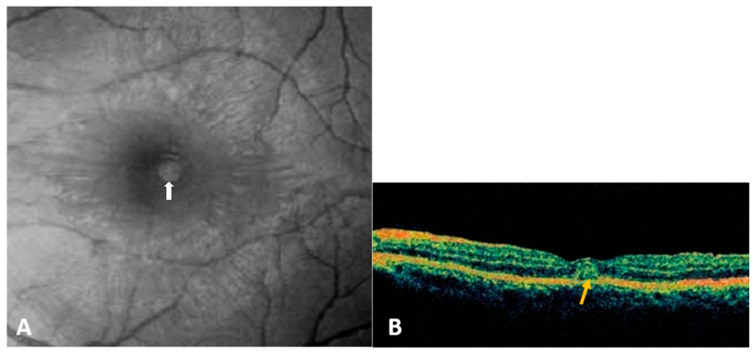
(**A**) Red-free fundus photograph of the left eye of a patient with dengue fever shows a subretinal round, yellowish lesion at the fovea (white arrow). (**B**) Optical coherence tomography through the lesion demonstrates conical foveal elevation (yellow arrow) with focal outer neurosensory retinal pigment epithelium thickening (Courtesy of Pr Soon Phaik Chee).

- Dengue-fever-associated maculopathy:
  DF-associated maculopathy, the most common ocular manifestation of acute DFV infection, is reported in 10% of hospitalized patients and is serotype-dependent [38,39]. Notably, one study linked maculopathy to serotype specificity, with a 10% incidence during DENV-1 epidemics but nothing during DENV-2 outbreaks [39]. Symptoms typically emerge 3–11 days after fever onset and improve over 2–4 weeks. Patients may present with sudden vision loss, central scotoma, or floaters. The lesions are typically asymmetric and often bilateral, primarily associated with intraretinal hemorrhages, manifesting as dots, blots, or flame-shaped hemorrhages. However, some patients remain asymptomatic, with lesions visible only through fluorescein or indocyanine green angiography (ICGA) [4,7,40,41]. Fluorescein angiography (FA) commonly reveals retinal vascular leakage (Figure 2) and occlusion, while ICGA identifies hypofluorescent spots corresponding to subretinal lesions and additional spots in areas without clinically evident dots [37]. OCT is valuable for detecting and monitoring dengue-induced inflammatory ischemic foveolitis and outer maculopathy, showing disruptions in outer retinal layers, conical foveal elevation, and focal thickening of the outer neurosensory retina RPE, aligning with round foveal yellowish lesions seen clinically (Figure 3). OCT is also instrumental in detecting and assessing associated serous retinal detachment (SRD) and macular edema. Teoh et al. used OCT to categorize patients into three groups: (1) diffuse retinal thickening, (2) cystoid macular edema, and (3) foveolitis. Their findings were correlated with visual acuity and prognosis [40]. DF-related foveolitis pertains to the yellow-orange central foveal lesion in patients with dengue maculopathy, visible on OCT as conical foveal elevation and focal outer neurosensory RPE thickening, often associated with prolonged scotomata persistence [40,42]. In dengue-related maculopathy, the prevalence of cystoid macular edema and foveolitis was evaluated to 24.6% and 33.7%, respectively [40]. More recently, OCT–angiography (OCTA) has revealed ischemia in the deep retinal capillary plexus [40,43].
- Other posterior segment manifestations

Other posterior ocular features associated with DF include posterior uveitis and, less commonly, vascular occlusions, panuveitis, vitritis, retinal and vitreous hemorrhages, choroidal changes, and yellow sub-retinal spots [6,37,42,44,45]. A study of patients (eyes, n = 65) with ocular manifestations of DF reported a prevalence of retinal vasculitis in 23.1% of cases [40].

- Anterior segment manifestations

In addition to the common subconjunctival hemorrhages observed in nearly half of the patients, reported anterior segment manifestations include anterior uveitis (7.7%) and, less frequently, shallow anterior chambers, acute angle-closure glaucoma due to ciliochoroidal effusion, superficial corneal punctate erosions, keratitis, and scleritis [7,36,42,46,47,48].

- Other ocular manifestations

There are rare reports of neuro-ophthalmic complications including optic disc swelling, neuroretinitis, neuromyelitis optica, and abducens palsy [42]. The reported rate of optic neuritis ranges from 0 to 1.5% [48]. A few cases of DF-associated panophthalmitis have been reported [49,50].

#### 3.1.4. Diagnosis

DF diagnosis relies on the characteristic clinical presentation and is confirmed through laboratory tests, including dengue virus nonstructural protein 1 detection and IgM/IgG antibody analysis [6,46]. Real-time RT-PCR or NS1 antigen detection is used for confirmation within the first 5 days, while ELISA-based detection of anti-dengue IgM or seroconversion in paired acute and convalescent serum samples are the more common methods for confirmation after 5 days [28]. Reported sensitivities of RT-PCR vary widely in the medical literature, ranging from 48.4% to 98.2% depending on sample timing [51]. Sensitivity is higher during the acute phase, coinciding with viremia. Additionally, sensitivity tends to be higher in primary infections compared to secondary infections, indicating the influence of the immune response [52]. RNA detection in urine samples extends beyond the viremic period by up to 16 days, with an estimated sensitivity ranging between 50% and 80% [53].

#### 3.1.5. Treatment and Prognosis

Treatment is essentially symptomatic [54]. In most cases, ocular manifestations resolve spontaneously with a favorable visual prognosis. However, severe cases have been treated with customized immunomodulatory therapies, including topical, periocular, oral, and intravenous steroids and immune globulins, with varying degrees of success depending on clinical presentation [41,55]. Various herbal remedies, such as *Carica papaya*, have been employed in treating DF [56]. A meta-analysis on *Carica papaya* indicated potential benefits in improving the platelet count during DF and reducing hospital stays, although the evidence quality was low [57]. The visual prognosis is good in most patients. However, permanent visual impairment may occur mainly due to retinal vasculitis, dengue maculopathy, or optic neuropathy [6].

Two live-attenuated tetravalent vaccines are currently available. Dengvaxia^®^ (Sanofi Pasteur, Chimeric viruses YFV/DEN1-4) is administered in three doses spaced six months apart. Its use is reserved for populations in which the seroprevalence is over 70% in the age bracket for vaccination, due to the weak efficacy of the vaccine and the potential long-term risks of severe DF in vaccinated seronegative subjects. The efficacy rate over the initial 25 months was assessed as 60.3% [58]. Vaccination is not recommended for children under 9 years of age [59]. More recently, Qdenga^®^ (Takeda, chimeric viruses DEN-2 PDK-53, DEN-1,-3,-4), administered in two doses with a 3-month interval, has received marketing authorization. The efficacy after two doses in children aged 4 to 16 years was assessed as 80.2% within 12 months [60]. At 18 months, the efficacy was lower, at 76.1% in immune recipients and 66.2% in non-immune recipients [61]. Vaccination can be performed in persons aged above 4, regardless of past dengue virus infection.

### 3.2. West Nile Virus (WNV) Infection

#### 3.2.1. Epidemiology

The West Nile virus (WNV), a zoonotic disease within the Japanese encephalitis serocomplex of viruses [62], has five distinct phylogenetic lineages. Among these, lineage one, which is globally distributed, and lineage two, primarily found in Africa, are known to cause human disease [62,63]. WNV primarily resides in birds, with transmission to humans occurring via *Culex* mosquitoes. Bird–mosquito–human transmission accounts for the majority of human cases [62]. The virus was first isolated in Uganda in 1937 and has since spread to Europe, Australia, Asia, and, since 1999, the United States, Canada, Mexico, the Caribbean, and parts of Central and South America [64]. Recently, co-circulation with the Usutu virus, another neurotropic mosquito-borne flavivirus, has been observed in Europe, raising the potential for WNV to expand to regions previously only affected by Usutu virus and vice versa [65].

#### 3.2.2. Systemic Manifestations

The incubation period for WNV infection ranges from 3 to 14 days and is most often followed in humans by an asymptomatic infection. Only approximately 25% of infected persons develop a self-limiting, non-specific viral illness, including fever, headaches, fatigue, nausea and vomiting, lymphadenopathy, and skin rashes, that typically lasts less than a week [62]. Severe neurologic manifestations may develop in less than 1% of cases including encephalitis, meningoencephalitis, acute flaccid paralysis, movement disorders, poliomyelitis-like syndrome, and Guillain–Barré Syndrome [63]. Asymmetric paralysis of acute onset and absence of reflexes without pain are characteristic of WNV [66]. Neuroinvasive disease is associated with high rates of morbidity and mortality, especially in patients with an advanced age or diabetes [66].

#### 3.2.3. Ocular Manifestations

Multifocal chorioretinitis, typically bilateral, is the most common ocular manifestation of acute WNV infection, occurring in nearly 80% of patients with neurologic symptoms [67,68]. However, the reported rate of multifocal chorioretinitis varies widely in the literature, ranging from 14% to 79% [69]. There appears to be an increased prevalence of multifocal chorioretinitis associated with the presence of neurological manifestations. [70]. WNV-associated chorioretinitis often presents with minimal symptoms, such as floaters, a mild vision reduction, redness, ocular pain, visual field defects, or diplopia [4,7,67,71,72].

**Figure 4:**
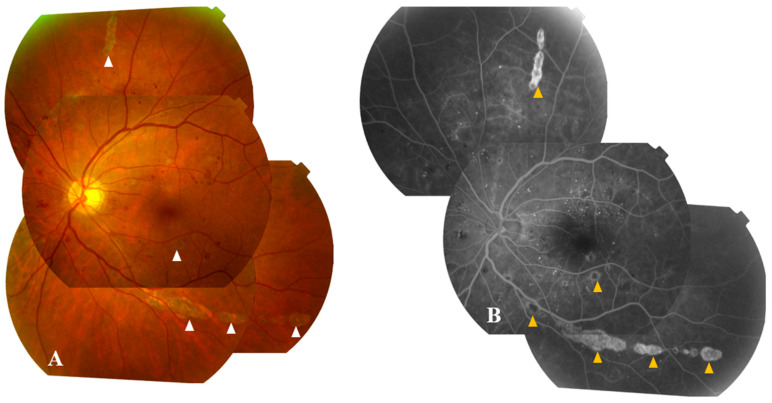
Color fundus photographs (**A**) and fluorescein angiograms (**B**) of the left eye of a diabetic patient with WNV infection show inactive multifocal chorioretinitis with typical linear clustering and the ‘target-like appearance’ of chorioretinal lesions (white arrowheads) with central hypofluorescence and peripheral hyperfluorescence (yellow arrowheads). Note the presence of associated moderate to severe non-proliferative diabetic retinopathy.

Active chorioretinal lesions are circular, deep, and yellowish on ophthalmoscopy, with early hypofluorescence and late staining on FA [67]. Inactive lesions appear round and atrophic, sometimes with central pigmentation, typically showing a “target-like appearance” on FA, featuring central hypofluorescence and peripheral hyperfluorescence (Figure 4) [67]. These lesions vary in number and size (ranging from 100 to 1500 µm in diameter), involving the midperiphery, possibly with posterior pole involvement [7,67]. They typically align radially in the nasal and peripheral fundus or follow a curvilinear pattern in the temporal posterior fundus, associated with the course of retinal nerve fibers [67,73].

OCT through lesions reveals their deep retinal location with focal disruption of the outer nuclear layer and retinal pigment epithelium (RPE) [74]. ICGA highlights well-defined hypofluorescent choroidal spots, often more numerous than clinically appreciated or seen on FA [75]. Chorioretinitis is more common in individuals over 50 and those with diabetes mellitus, with a significant proportion also displaying concurrent diabetic retinopathy.

Other manifestations have been described, including non-granulomatous anterior uveitis, retinal hemorrhages, focal or diffuse retinal vascular sheathing, vascular leakage, occlusive vasculitis, congenital chorioretinal scarring, zones of atrophy and mottling of the RPE, macular edema, optic neuritis, papilledema, and binocular diplopia related to sixth cranial nerve involvement [6,7,44,67,68,72,76,77]. Patients (n = 51 eyes) with intraocular inflammation tested positive for WNV-related vitritis (73%), papilledema (14%), intraretinal hemorrhages (43%), vasculitis (32%), and retinal occlusive vasculitis (16%) [71].

Noteworthy, OCTA allows for the detection and precise delineation of areas of retinal capillary nonperfusion in both the superficial and deep capillary plexuses in cases of associated occlusive retinal vasculitis [78].

#### 3.2.4. Diagnosis

WNV infection is diagnosed primarily based on clinical features and confirmed through the detection of IgM antibodies in serum or cerebrospinal fluid [79]. However, serological tests may show cross-reactivity with other flaviviruses or yield false negative results [25]. Therefore, virus detection using RT-PCR is increasingly becoming the gold standard for WNV diagnosis [79]. During the viremic period, RT-PCR sensitivity was evaluated at 86.8% in whole-blood samples [80]. Detection rates were lower in serum, cerebrospinal fluid, plasma, and urine, respectively, at 26%, 16.6%, 20%, and 58.3% [80].

#### 3.2.5. Treatment and Prognosis

Neurological manifestations are addressed through intensive supportive care. Ongoing clinical trials are exploring novel therapeutic strategies, including interferon alpha-2b, interferon beta, and high-titer intravenous immunoglobulin [81,82,83]. Potential in vitro antiviral compounds include Delphinidin and Epigallocatechin Gallate [84]. The primary approach to WNV infection control remains prevention, achieved by reducing mosquito populations (such as draining standing water and using larvicides) and implementing personal protection measures (such as repellents, window screens, and protective clothing) [62]. Four veterinary vaccines are approved for use in horses, requiring annual boosters [85]. Currently, there are no licensed human vaccines available. Only phase 2 clinical trials have been conducted [86]. A live attenuated chimeric vaccine (ChimeriVax WN02) elicited immunogenicity after a single dose. A predictive model indicated that effective WNV vaccination in areas with increased incidence could potentially reduce the annual morbidity of neuroinvasive diseases cases by 30% and deaths by 60% in the United States [87].

Specific ophthalmic treatments may be necessary to address intraocular inflammation or posterior segment complications. These treatments include topical steroids for anterior uveitis, peripheral retinal photocoagulation for neovascularization resulting from occlusive vasculitis, pars plana vitrectomy for non-clearing vitreous hemorrhage or tractional retinal detachment, and intravitreal injection of anti-vascular endothelial growth factor agents for choroidal neovascularization or associated macular edema in cases of chorioretinitis [88,89,90]. The benefit of systemic steroids is not evidence-based, but the positive role in recovery could justify their use in cases of severe posterior segment involvement. Ocular diseases associated with WNV infection usually have a self-limited course, and visual acuity returns to baseline in most patients [67]. However, persistent visual impairment can occur due to various factors, including chorioretinal scarring, choroidal neovascularization, vitreous hemorrhage, tractional retinal detachment, severe ischemic maculopathy, optic atrophy, and retrogeniculate damage [6,44,67,68,76,91,92].

### 3.3. Yellow Fever Virus

#### 3.3.1. Epidemiology

Yellow fever virus (YFV) is primarily transmitted to humans through bites from infected *Aedes aegypti* and *Aedes albopictus* mosquitoes. It is maintained through sylvatic (jungle) transmission cycles involving mosquitoes and non-human primates, as well as mosquito–human transmission cycles in urban areas [93,94]. YFV is a re-emerging arboviral disease that can potentially be lethal, causing an estimated 30,000–60,000 fatalities annually in endemic regions across Africa, South, and Central America [95].

#### 3.3.2. Systemic Manifestations

Yellow fever is characterized by rapid jaundice development and liver dysfunction, which are distinctive clinical features of the disease. Most YFV-infected individuals are either asymptomatic or experience an acute febrile phase lasting around 4 days, accompanied by symptoms like myalgia, headaches, back pain, nausea, and vomiting. This phase typically resolves within days. However, approximately 15–25% of patients progress to a severe toxic form of the disease, which includes symptoms such as viremia, fever, jaundice, prostration, hematemesis, hemorrhagic diathesis, and failure of the liver, kidneys, and myocardium. This severe form is associated with a 50% mortality rate [93].

#### 3.3.3. Ocular Manifestations

In the initial phase of yellow fever, conjunctivitis is the most common ocular finding. However, scleral icterus often occurs during the toxic phase [4,7]. Some case reports have suggested additional ocular manifestations. In yellow fever patients, retinopathy may occur in 20% of cases [96], which includes retinal nerve fiber layer infarcts (55%), superficial hemorrhages (35%), and grayish deep lesions (30%). Silvana Vianello et al. documented two intensive care patients in Brazil who exhibited increased choroidal thickness bilaterally, with retinal vein congestion in one patient and a 360° mid-peripheral choroidal detachment with yellowish subretinal lesions in the other patient [97]. Another publication reported the case of a 53-year-old Brazilian patient with a YFV infection with a unilateral retinal edema, macular exudates, and hemorrhages during the convalescent stage, possibly due to an immune-mediated mechanism rather than direct viral invasion or infection of the ocular tissues [97].

#### 3.3.4. Diagnosis

Diagnosis of YFV is based on PCR of blood or urine samples in the early stages, and ELISA or PRNT antibody detection in later stages [93,97]. Data regarding PCR sensitivities are scarce. A Brazilian study conducted after the last outbreak from 2016 to 2018 indicated detection rates of 8.9% in serum and 50% in blood samples during the viremic phase [98]. Urine sample sensitivity was evaluated as 25% [99]. The RNA recovery in urine samples could extend up to 69 days after symptom onset [99].

#### 3.3.5. Treatment and Prognosis

The development of two live-attenuated YF vaccines in the 1930s and their wide deployment in the 1940s led to a significant decline in the disease. The historical 17 D strain is still used to produce the vaccine. A meta-analysis confirmed a high seroprotection rate after a single dose which lasted five to ten years after vaccination [100]. However, some ocular complications related to YFV vaccination have been reported, particularly when the live YFV vaccine is administered alongside other vaccines such as Hepatitis A/B, *Neisseria Meningitidis*, or Typhoid [7]. These complications vary and encompass anterior and intermediate uveitis, unilateral optic neuropathy, multifocal choroiditis, evanescent white dot syndrome, arteriolar occlusion, and conditions resembling Vogt–Koyanagi–Harada disease [4,101,102]. Other YF vaccine candidates are currently in development and have not yet been tested beyond phase 1 clinical trials [103].

There is currently no proven specific antiviral drug available, with a strong emphasis on prevention through public health measures and vaccination [104,105]. Ongoing clinical trials are investigating novel therapeutic strategies, including phase I human immunoglobulin anti-YFV [106]. A Brazilian randomized clinical trial is assessing the efficacy of the antiviral sofosbuvir, a nucleotide analogue inhibitor, that exhibits activity in vitro and in animal models [107]. Ribavirin has shown potential activity in vitro but requires high concentrations that are not achievable in human serum [108]. In cases of severe systemic disease, intensive supportive therapy is indicated. Ocular complications associated with YFV and its vaccine are most often self-limiting, with a favorable course with supportive care.

### 3.4. Zika Virus

#### 3.4.1. Epidemiology

Zika virus is primarily transmitted by *Aedes* mosquitoes [109], but it can also be transmitted vertically during pregnancy [22], through sexual contact [110], and via contaminated blood transfusions [111]. The first human case was reported in Uganda, Africa, in 1952 [112], and subsequent epidemics have occurred globally since 2007, including in Micronesia, French Polynesia, and South America [109]. In 2016, a significant outbreak occurred in Brazil, affecting 1.6 million people, with 1950 cases of infection-related microcephaly in newborns [113]. The infection can pose a substantial burden, especially in infants with congenital Zika syndrome (CZS) and adult patients with Guillain–Barre syndrome.

#### 3.4.2. Systemic Manifestations

In adults, Zika infection is asymptomatic in 80% of cases [114]. After an incubation of 3–12 days, infected patients experience nonspecific flu-like symptoms [109]. Neurological complications may occur, including encephalitis, myelitis, and Guillain–Barre syndrome [115]. Generally, the median time between the onset of the infectious disease and neurologic features varies between 5 and 12 days [116]. Intrauterine infection and maternal–fetal transmission can occur during pregnancy in patients with a Zika infection. In newborns, CZS is defined by a pattern of anomalies including microcephaly, brain abnormalities, osteoskeletal abnormalities (mainly arthrogryposis and club foot), neurosensorial defects, and ocular manifestations (see below) [117].

#### 3.4.3. Ocular Manifestations

In the acute phase, reported ophthalmological signs include non-purulent conjunctivitis (40%) and retroorbital pain (40%) [8,118,119]. Mild non-granulomatous anterior uveitis may be present in approximately half of patients presenting with red eyes [118]. Less-frequent manifestations have been described. Acute maculopathy has been reported in a 64-year-old man. OCT showed a disruption of the outer retinal layer in the central macula [120]. Otherwise, bilateral posterior uveitis with chorioretinal lesions was described in a 26-year-old man [121]. OCT showed hyperreflective nodules in the outer retina, and indocyanine green angiography confirmed active choroidal lesions.

**Figure 5:**
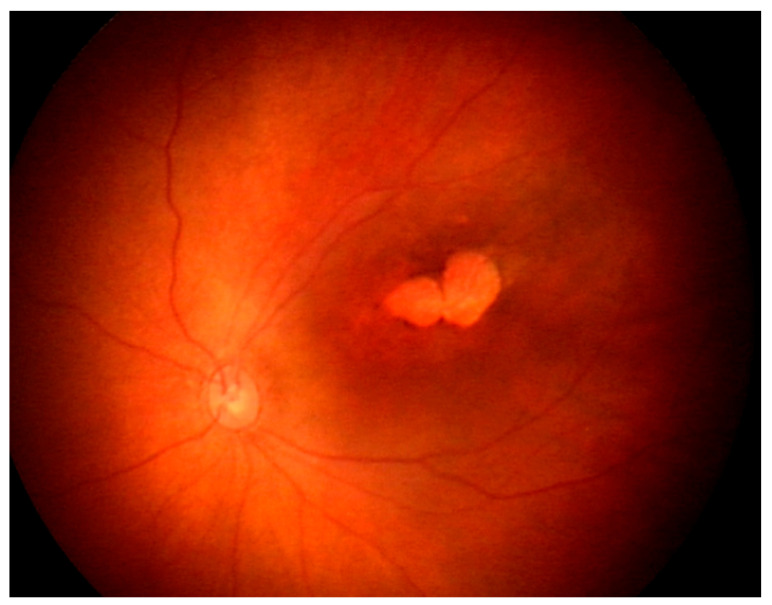
Fundus photograph of the left eye of a newborn with congenital Zika virus syndrome shows macular, well-delineated atrophic lesions (Courtesy of Pr Rubens Belfort).

Ocular findings have been observed in 21.4–55% of CZS newborns [122]. Risk factors for ocular involvement in CZS include a smaller cephalic diameter at birth and infective symptoms during the first trimester of pregnancy [123]. However, ocular findings may occur in infants without microcephaly, suggesting that the absence of this feature does not preclude CZS screening. The most common ocular signs (85%) are macular and optic nerve abnormalities [124]. Posterior segment lesions include chorioretinal atrophy (Figure 5) and pigment mottling in the macular area [125,126]. Optical coherence tomography (OCT) of these lesions shows ellipsoid zone disruption (100%) and retinal and choroidal thinning (respectively, 89% and 78%) [127]. Retinal hemorrhages can also be observed on ocular fundus and are considered a screening criterion for CZS [128]. Anterior segment lesions include iris coloboma, corneal ectasia, lens subluxation, cataracts, and congenital glaucoma (12%) [129,130,131]. Neuroophthalmological lesions encompass strabismus, disc hypoplasia, increased cup-to-disc ratios, disc pallor, and horizontal nystagmus [125]. Histopathological studies on deceased fetuses have revealed pupillary membranes, immature anterior chamber angles, loss of pigment, retinal pigment epithelium thinning, choroidal thinning, and undifferentiated nuclear layers of the retina [132].

#### 3.4.4. Diagnosis

A biological diagnosis can be obtained using various samples, including urine, amniotic fluid, serum, cerebrospinal fluid, semen, and tears, employing molecular biology techniques such as RT-PCR and serology methods like ELISAs [109,133]. Molecular diagnosis is typically carried out during the acute phase of the disease, approximately 3–5 days after incubation [133]. The rate of RNA detection in serum samples is low, ranging from 41% to 56% within 5 days of symptom onset [134,135,136], while in urine samples, it is higher, at around 95% [136]. Serial serum sampling between days 3 and 13 can detect an additional 25% of cases [134]. RNA detection in urine samples can remain positive for up to three weeks [137]. IgM antibodies can be detected in serum and cerebrospinal fluid samples within the first week of illness [138]. If IgM results are equivocal, confirmation can be obtained using a PRNT [133,138].

#### 3.4.5. Treatment and Prognosis

No specific treatment is currently available. However, natural products isolated from medicinal herbs have demonstrated inhibition of ZIKV in vitro [139]. Only one in vivo animal model is available, reporting the anti-ZIKV activity of emetine [140].

Well-tolerated vaccine candidates have progressed through phase 1 or phase 2 development [141]. A completed phase 2/2B trial assessed a ZIKA DNA vaccine (VRC 5283) expressing prM-E structural genes. This vaccine was immunogenic in humans and generated cross-reactivity but did not produce cross-neutralizing antibodies against other flaviviruses, especially DENV [142]. Another ongoing phase 2 trial is evaluating a ZIKA mRNA vaccine (mRNA-1853) delivering modified prM-E mRNA [143]. However, the lack of current outbreaks hinders the advancement of clinical trials. Supportive care is recommended for adult patients experiencing inflammatory ocular manifestations. Corticosteroid eye drops can be employed to manage anterior uveitis [118].

The burden of Zika virus infection can be substantial, particularly in cases of congenital Zika syndrome (CZS). Ophthalmologists play a crucial role in early CZS assessment in newborns and the treatment of visual impairments and related ocular issues. Recommendations from the French High Council for Public Health [144] and the CDC [145] advise comprehensive ophthalmic screenings for all infants born from mothers infected during pregnancy, with the first examination before one month of age and a follow-up at one year. Public health campaigns should reinforce preventive measures, including the use of repellents to prevent mosquito bites and advising pregnant women in their first two trimesters to avoid traveling to disease-endemic areas [133].

### 3.5. Japanese Encephalitis Virus

#### 3.5.1. Epidemiology

Japanese encephalitis (JE), caused by the JEV, is predominantly found in Asia and the Western Pacific, including Australia, where outbreaks have been reported recently [146]. Annually, an estimated 50,000 cases of JE are reported globally, resulting in approximately 15,000 deaths [147]. JE primarily occurs in rural areas and is transmitted through an enzootic cycle involving *Culex* mosquitoes and vertebrate hosts, including water birds and swine [148]. Vertical transmission has also been documented [21].

#### 3.5.2. Systemic Manifestations

After an incubation period of 5 to 15 days [149], infected individuals develop flu-like symptoms with fever. Neurological complications, such as encephalitis, occur in only 1 out of 200–300 infected individuals [150]. During the encephalitic phase, various neurological symptoms may arise depending on the anatomical lesions. JEV infection can lead to a Parkinsonian syndrome or polio-like acute flaccid paralysis [151]. The case fatality rate is estimated to be as high as 30% among patients with neurological complications [152]. Survivors may experience a high burden of the disease, with 30–50% developing long-term sequelae [152].

#### 3.5.3. Ocular Manifestations

Reports of ocular complications related to JEV are rare and likely underdiagnosed. One case occurred in a 53-year-old woman in 2006, who developed ischemic maculopathy during infection [153]. Fundus examination revealed a “white” macula and retinal hemorrhages, while FA showed non-perfusion areas of the macula. Another case was reported in 2020, involving a 45-year-old man who had visited Bali [154]. He experienced febrile confusion with dysphasia and bilateral conjunctival injection. Ophthalmological examination revealed moderate non-granulomatous anterior uveitis, and the ocular fundus showed bilateral vessel tortuosity and pale chorioretinal lesions, suggestive of chorioretinitis [154].

#### 3.5.4. Diagnosis

Available diagnostic tests include serology (antibody capture ELISA) and RT-PCR, performed on serum and cerebrospinal fluid samples [27]. The gold standard for diagnosing JEV is IgM testing by ELISA in cerebrospinal fluid, with specific IgM detectable during the first week of illness [155]. Cross-reactions are common among flaviviruses, which is concerning in regions where both the dengue virus and JEV are present. The sensitivity of molecular diagnosis is low, at less than 25% in the acute phase [156,157]. Combining the two techniques can increase the sensitivity to 31% [158].

#### 3.5.5. Treatment and Prognosis

JEV infection can lead to severe systemic disease, including neurological manifestations which can be managed with supportive care. Potential in vitro antiviral compounds include Baicalein, Curcumin, and Kaempferol [84]. Specific therapies are inadequate, with corticosteroids, ribavirin, minocycline, intravenous immunoglobulin, and interferon alpha-2a showing inefficacy [151]. However, vaccination has reduced the burden of JEV disease [159]. Currently available vaccines include cell-culture-derived live-attenuated; cell-culture-derived killed-inactivated; and cell-culture-derived live-attenuated chimeric vaccines [160]. The use of mouse-brain-derived killed-inactivated vaccines, which induced immune adverse events, has been discontinued [160]. Most vaccines are based on the SA14-14-2 strain, providing nearly complete protection with two doses for up to five years [151]. It is advisable, according to the CDC’s advisory committee on immunization practices, to recommend JEV vaccination for long-term travel to endemic regions, or for persons moving to an endemic country to live [149]. The JEV vaccine can also be considered in residents of rural areas in endemic locations. Indeed, implementation of vaccination programs reduced the incidence of JEV diseases in some countries [161]. The scarcity of reports on ocular manifestations hinders definitive conclusions about visual prognosis of JEV-related ocular issues. Nevertheless, cases of severe ischemic macular involvement indicate a potentially high burden of ocular complications [153].

### 3.6. Kyasanur Forest Disease Virus

#### 3.6.1. Epidemiology

Kyasanur forest disease virus (KFDV) is a biosafety level 4 organism belonging to the tick-borne encephalitis serocomplex, causing a rare hemorrhagic fever disease currently limited to India [162]. First reported in 1957 in Kyasanur Forest, Karnataka, India [163], there have been about 500 annual cases in recent decades [164]. KFDV is transmitted to humans and animals through the bite of an infected hard tick (*Hemaphysalis turturis*/*spinigera*) or contact with an infected animal [165]. Variants of KFDV have been identified in patients with hemorrhagic fever in Saudi Arabia, referred to as Alkhurma hemorrhagic fever disease, sharing 97% homology with KFDV and suggesting a common ancestral origin [162].

#### 3.6.2. Systemic Manifestations

KFD typically follows a biphasic course. After an incubation period of 2–7 days, patients experience an acute febrile phase characterized by flu-like symptoms and hemorrhagic manifestations, lasting up to two weeks [162]. In the convalescent phase, 10–20% of patients may experience severe hemorrhagic fever symptoms, including gastrointestinal bleeding and pneumonia, along with neurological complications resembling meningoencephalitis [162]. The estimated case fatality rate ranges from 3 to 10% [166].

#### 3.6.3. Ocular Manifestations

Ocular manifestations are common in KFD, with rare historical studies reporting conjunctival congestion as the most frequent ocular sign (100%) [167]. Other ophthalmic features include hemorrhages in various ocular sites (conjunctiva (11%), vitreous humor (1%), retina (12%)), iritis (3%), keratitis (9%), and papilledema (1%) that may occur in encephalitic patients [167]. Lens opacification has been reported [168].

#### 3.6.4. Diagnosis

Biological diagnosis can be performed from serum samples in the early stage (first two weeks) using RT-PCR [169]. Serology (antibody capture ELISA) can be used in the acute (IgM) and convalescent phases (IgG) [169]. Studies assessing the diagnostic performance of RT-PCR are limited. However, one study demonstrated a low detection rate of approximately 34.3% in serum samples [169].

#### 3.6.5. Treatment and Prognosis

Specific therapies are deficient; notably, inefficacy was observed with interferon alpha-2a [170]. A vaccination strategy was developed in the 1990s using a formalin-inactivated tissue culture vaccine [171]. The vaccine’s efficacy is estimated at 83% after two doses. Annual administration of booster doses is recommended for five years after the last report of KFD cases in the area [172]. Currently, efforts are ongoing to optimize this vaccine by reducing the formalin concentration to mitigate side effects such as swelling and irritation, aiming to enhance vaccine acceptance [173]. Public health campaigns in affected areas promote the use of repellents to prevent tick bites [162]. Limited data are available on the visual outcomes of this rare disease, which mainly occurs in low-resource rural areas [162].

## 4. Conclusions

Flavivirus infections are on the rise due to various human activities, including climate change and deforestation, which promote vector-borne zoonotic diseases [174,175]. This emergence represents a potential public health issue, resulting in not only conditions that can be life-threatening but also those that can jeopardize vision. This was illustrated in recent Zika virus outbreaks that have resulted in substantial ocular morbidity [113]. In accordance with the information provided in this review, most cases of human flavivirus infections are linked to eye-related symptoms, which might be inaugural. Nevertheless, these symptoms can vary widely. On one hand, certain clinical features can be distinctive and indicative of the infection, such as the linear pattern of chorioretinitis seen in WNV cases. On the other hand, some viruses can cause non-specific symptoms, making diagnosis challenging. The screening for flavivirus infections should be tailored to the specific clinical context, taking into consideration epidemiological factors like current outbreaks and recent travel history. Additionally, a thorough ophthalmologic evaluation seems crucial if visual symptoms develop during the course of a flavivirus infection.
